# Neoadjuvant and Concurrent Chemotherapy Have Varied Impacts on the Prognosis of Patients with the Ascending and Descending Types of Nasopharyngeal Carcinoma Treated with Intensity-Modulated Radiotherapy

**DOI:** 10.1371/journal.pone.0161878

**Published:** 2016-10-26

**Authors:** Ji-Jin Yao, Guan-Qun Zhou, Fan Zhang, Wang-Jian Zhang, Li Lin, Ling-Long Tang, Yan-Ping Mao, Jun Ma, Ying Sun

**Affiliations:** 1 Department of Radiation Oncology, Sun Yat-sen University Cancer Centre, State Key Laboratory of Oncology in South China, Collaborative Innovation Centre for Cancer Medicine, Guangzhou 510060, Guangdong Province, People's Republic of China; 2 Department of Radiation Oncology, the Fifth Affiliated Hospital of Sun Yat-sen University, Zhuhai 519001, Guangdong Province, China; 3 Department of Medical Statistics and Epidemiology & Health Information Research Centre & Guangdong Key Laboratory of Medicine, School of Public Health, Sun Yat-sen University, Guangzhou 510080, Guangdong Province, China; Shanghai Jiao Tong University School of Medicine, CHINA

## Abstract

**Purpose:**

To compare the outcomes of patients with ascending type (T4&N0-1) and descending type (T1-2&N3) of nasopharyngeal carcinoma (NPC) treated with concurrent chemoradiotherapy (CCRT), neoadjuvant chemotherapy (NACT) + intensity-modulated radiotherapy (RT) or NACT + CCRT.

**Methods:**

Retrospective analysis of 839 patients with ascending or descending types of NPC treated at a single institution between October 2009 to February 2012. CCRT was delivered to 236 patients, NACT + RT to 302 patients, and NACT + CCRT to 301 patients.

**Results:**

The 4-year overall survival rate, distant metastasis-free survival rate, local relapse-free survival rate, nodal relapse-free survival rate, loco-regional relapse-free survival rate, and progression free survival rate were 75.2% and 73.4% (P = 0.114), 85.7% and 74.1% (P = 0.008), 88.8% and 97.1% (P = 0.013), 96.9% and 94.1% (P = 0.122), 86.9% and 91.2% (P = 0.384), 73.7% and 66.2% (P = 0.063) in ascending type and descending type. Subgroup analyses indicated that NACT + RT significantly improved distant metastasis-free survival rate and progression-free survival rate when compared with CCRT in the ascending type, and there were no significant differences between the survival curves of NACT +RT and NACT + CCRT. For descending type, there were no significant differences among the survival curves of NACT +RT, CCRT, and NACT + CCRT groups, and the survival benefit mainly came from CCRT.

**Conclusions:**

Compared with NACT + CCRT or CCRT, NACT + RT may be a reasonable approach for ascending type. Although concurrent chemotherapy was effective in descending type, NACT + CCRT may be a more appropriate strategy for descending type.

## Introduction

Long-term loco-regional control and overall survival (OS) in early-stage nasopharyngeal carcinoma (NPC) exceeded 95% after the introduction of intensity-modulated radiotherapy (IMRT) [1]. However, the 5-year OS rate for locoregionally-advanced stage declines to 41–63% [2–4]; therefore, treatment outcomes for advanced NPC are necessary to improve.

Several publications [5–6] and meta-analyses [7–8] reported concurrent chemotherapy provides the largest survival benefit. Some clinical trials and meta-analyses [9–10] demonstrated neoadjuvant chemotherapy (NACT) followed by concurrent chemoradiotherapy (CCRT) was well-tolerated and provided good outcomes, while others [11–13] question the value of concurrent chemotherapy in patients with locoregionally-advanced NPC (LA-NPC) treated with IMRT. These discrepancies may be due to the heterogeneity of LA-NPC. As reported by Wee *et al*. [14], NPC patients with predominantly advanced local disease (advanced T stage: T3-4) and early-stage cervical lymph-node involvement (early N stage: N0-1) were classified as having the ascending-type of the disease, which usually experienced local failure; whereas those with advanced lymph-node metastases (advanced N stage: N2-3) and early-stage local disease (early T stage: T1-2) were classified as having the descending type, which distant failure was more common. There two types of NPC could exhibit distinct clinical-biological behaviors. However, previous studies did not take tumour heterogeneity into account for the survival analysis. On the basis of premise, we hypothesized that the proportion of the ascending and descending types may directly affect the conclusions of research.

To test this hypothesis, we conducted a retrospective study to compare the efficacy of neoadjuvant and concurrent chemotherapy in patients with ascending and descending types of LA-NPC.

## Materials and Methods

### Patient characteristics and treatment

The inclusion criteria for this study were as follows:

1. Histology: histologically-proven undifferentiated or non-keratinizing squamous cell carcinoma of the nasopharynx.
2. Stage: ascending (T4&N0-1) or descending (T1-2&N3) NPC with no evidence of distant metastasis. All patients were staged using the 7^th^ UICC/AJCC staging system [15]. Tumor staging was based on routine examination (physical examination, nasopharyngeal fiberoptic endoscopy, chest X-ray, abdominal sonography, magnetic resonance imaging, bone scan, positron emission tomography-computed tomography).
3. Treatment modality: Patients treated using radical IMRT with neoadjuvant or/and concurrent chemotherapy; patients who received adjuvant chemotherapy were excluded.
4. Radiotherapy: Target volumes were delineated according to our institutional treatment protocol [16], in agreement with International Commission on Radiation Units and Measurements Reports (ICRU) 50 and 62. Planning target volumes (PTVs) for all gross tumour volumes and clinical target volumes (CTVs) were generated automatically after tumour target delineation according to immobilization and localization uncertainties. Clinical target volumes (CTV) were individually delineated based on tumor invasion patterns. The prescribed dose was a total dose of 68–70 Gy at 2.12–2.27 Gy/fraction to planning target volume (PTV) of GTV-P, 60 Gy to PTV of CTV-1 (i.e., high-risk regions) and 54 Gy to PTV of CTV-2 (i.e., low-risk regions). All patients received one fraction daily, five days per week. All patients have no additional boosts and were treated with radical intent.
5. Chemotherapy: Neoadjuvant chemotherapy regimens were TP (135 mg/m^2^ paclitaxel intravenous injection [I.V.] on day 1; 80 mg/m^2^ cisplatin I.V. on day 1) or PF (80 mg/m^2^ cisplatin I.V. on day 1; 800 mg/m2/d 5-fluorouracil, continuous I.V. infusion on days 1–5) every three weeks for 3 cycles. Concurrent chemotherapy was 80–100 mg/m^2^ cisplatin I.V. every three weeks for 3 cycles or 30–50 mg/m^2^ cisplatin I.V. weekly for 6 cycles. Deviations from institutional guidelines were due to organ dysfunction (suggesting intolerance to chemotherapy) or patient refusal.

All patients that treated at Sun Yat-sen University Cancer Center (SYSUCC) between October 2009 to February 2012 were assessed using these criteria. In total, 839 patients fulfilled all of the criteria above and were included in this retrospective analysis. The Ethics committee of SYSUCC also waived the need for written consent because this was a retrospective study; verbal consent was obtained from the patients via telephone and documented in the informed consent form if the patient agreed to participate in this study.

### End-points and statistical analysis

The following endpoints (time to the first defining event) were assessed: overall survival (OS), distant metastasis-free survival (DMFS), local relapse-free survival (LRFS), nodal relapse-free survival (NRFS), loco-regional relapse-free survival (LRRFS) and progression free survival (PFS). The OS was defined as the time from diagnosis of NPC to death from tumor. DMFS was defined as the time from the date of treatment to the date of the first observation of a distant metastases. LRFS was defined as the time from the diagnosis of NPC to the absence of a primary site relapse. NRFS was defined as the time from the diagnosis of NPC to the absence of a neck lymph node relapse. LRRFS was defined as the time from the diagnosis of NPC to the absence of a primary site or neck lymph node relapse. PFS was defined as the time from the diagnosis of NPC to events that included death or disease progression at local, regional, or distant sites.

All analyses were performed using R3.1.2. Fisher’s exact test was used to compare the basic characteristics of the patients among different treatment groups. The Kaplan–Meier method was used to analyze the time-to event endpoints, and the log-rank test was used to compare the survival curves among different treatment groups. All statistical tests were two-sided; P < 0.05 was considered statistically significant.

### Follow-up

Patients were assessed every 3 months in first 2 years, every 6 months in second to fifth years and annually thereafter. Patients who returned to the clinic received a series of examinations: blood biochemical analysis, nasopharyngeal fiberoptic endoscopy, chest X-ray, abdominal sonography and MRI. Bone scans and PET-CT were performed for patients with suspected metastases. If patients did not return to the clinic, follow-up information from the patients themselves, their families, or the household registration office was obtained mainly by phone. Follow-up was calculated from first day of therapy to death or last examination. Median follow-up for the ascending type was 50.4 months (range, 5.9–72.3 months) and 49.8 months (5.3–71.2 months) for the descending type.

## Results

### Patient characteristics

Between October 2009 to February 2012, clinical data of 839 NPC patients treated in SYSUCC who met all of the criteria were retrospectively analyzed. Of these patients, 451 patients were classified as ascending type and 388 were classified as descending type. Of the 839 patients, CCRT was delivered to 236 (28.1%) patients, NACT + RT to 302 (36.0%), and NACT + CCRT to 301 (35.9%). The male: female ratio was 2.7:1 (611 men and 228 women), and the median age was 45 years (range, 11–74 years). Histological examination revealed that 99.3% of patients had WHO II/III disease, 0.7% had WHO I disease. No significant differences were found between these groups in baseline characteristics (Table 1).

**Table 1:** Basic characteristics of the patients in the CCRT, NACT + IMRT and NACT + CCRT groups stratified by the ascending and descending types of NPC.

	Ascending type [n, (%)]				Descending type [n, (%)]			
Characteristic	CCRT	NACT + RT	NACT + CCRT	*P* *	CCRT	NACT + RT	NACT + CCRT	*P* *
**Total**	146 (32.4)	164 (36.4)	141 (31.3)		90 (23.2)	138 (35.6)	160 (41.2)	
**KPS**	90 ± 6	87 ± 8	85 ± 9	0.786	93 ± 5	90 ± 7	88 ± 7	0.823
**Age (years)**				0.545				0.769
< 45	53 (36.3)	54 (32.9)	46 (32.6)		35 (38.9)	51 (37.0)	62 (38.8)	
≥ 45	93 (63.7)	110 (67.1)	95 (67.4)		54 (61.1)	87 (63.0)	98 (61.2)	
**Gender**				0.735				0.442
Male	112 (76.7)	125 (76.2)	103 (73.0)		58 (64.4)	99 (71.7)	114 (71.3)	
Female	34 (23.3)	39 (23.8)	38 (27.0)		32 (35.6)	39 (28.3)	46 (28.7)	
**Histology**				0.994				0.912
WHO I	1 (0.7)	1 (0.6)	1 (0.7)		1 (1.1)	1 (0.7)	1 (0.6)	
WHO II/III	145 (99.3)	163 (99.4)	140 (99.3)		89 (98.9)	137 (99.2)	159 (99.4)	
**T category**				-				0.788
T1	0 (0)	0 (0)	0 (0)		32 (35.6)	50 (36.2)	63 (39.4)	
T2	0 (0)	0 (0)	0 (0)		58 (64.4)	88 (63.8)	97 (60.6)	
T4	146 (100)	164 (100)	141 (100)		0 (0)	0 (0)	0 (0)	
**N category**				0.745				-
N0	30 (20.5)	35 (21.3)	34 (20.6)		0 (0)	0 (0)	0 (0)	
N1	116 (79.5)	129 (78.7)	107 (79.4)		0 (0)	0 (0)	0 (0)	
N3	0 (0)	0 (0)	0 (0)		90 (100)	138 (100)	160 (100)	

*Abbreviations*: Ascending type, patients stage with T4N0-1M0; descending type, patients stage with T1-2N3M0; WHO: World Health Organization; NACT: neoadjuvant chemotherapy; RT: radiotherapy; CCRT: concurrent chemoradiotherapy.

* *P* values were calculated using Fisher’s exact test.

### Treatment compliance

All patients completed the full course of radiotherapy. In the NACT + RT group, all patients completed two cycles of NACT at least. In the NACT + CCRT group, all patients completed two cycles of NACT and two cycles of concurrent chemotherapy at least. In the CCRT group, all patients completed two cycles of concurrent chemotherapy (every three weeks) or four cycles of concurrent chemotherapy (weekly) at least.

### Treatment outcomes

The 4-year OS rate, DMFS rate, LRFS rate, NRFS rate, LRRFS survival rate, and PFS rate were 75.2% and 73.4% (P = 0.114), 85.7% and 74.1% (P = 0.008), 88.8% and 97.1% (P = 0.013), 96.9% and 94.1% (P = 0.122), 86.9% and 91.2% (P = 0.384), 73.7% and 66.2% (P = 0.063) in ascending type and descending type. In addition, patients with ascending type had significantly better DMFS than patients with descending type, but patients with descending type had significantly better LRFS than patients with ascending type. Patients treated with chemoradiotherapy were divided into three groups: CCRT group, NACT + RT group, and CCRT + CCRT group.

**Fig 1:**
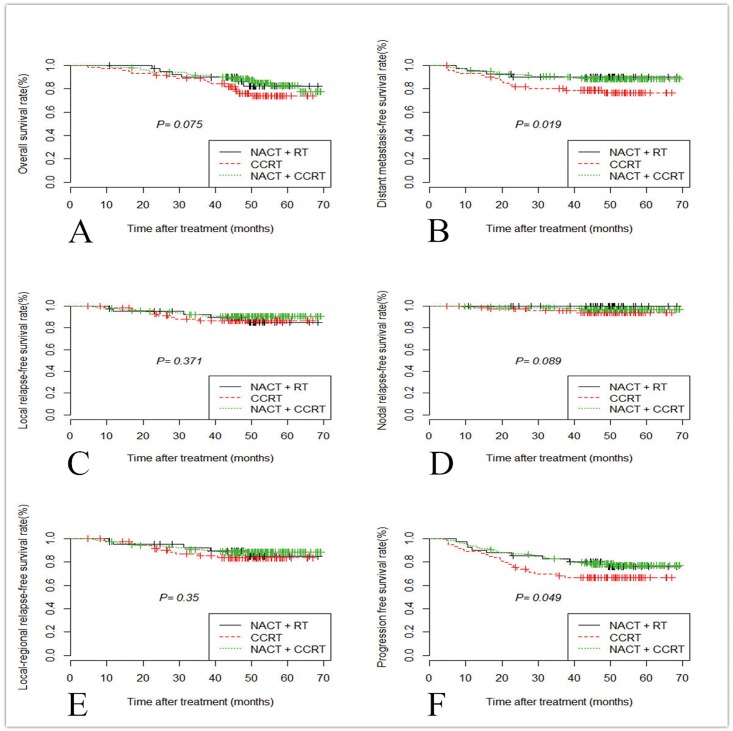
Kaplan-Meier curves of overall survival (A), distant metastasis-free survival (B), local relapse-free survival (C), nodal relapse-free survival (D); loco-regional relapse-free survival (E) and progression-free survival (F) in patients with ascending type (T4&N0-1) treated with NACT + RT, CCRT, and NACT + CCRT. *Abbreviations*: NACT: neoadjuvant chemotherapy; RT: radiotherapy; CCRT: concurrent chemoradiotherapy.

For ascending type, the 4-year OS rates of the three chemoradiotherapy groups were 76.7%, 85.4%, and 84.4%, respectively. Compared with CCRT alone, OS of other groups were significantly higher. But there were no statistically significant difference among these three groups (Fig 1A). The 4-year DMFS rates of the three chemoradiotherapy groups were 78.1%, 90.2%, and 88.7%, respectively. Compared with CCRT alone, NACR + RT and NACT + CCRT improved DMFS significantly (Fig 1B, P = 0.019). Four-year LRFS rate for ascending type was 87.7% after CCRT, 87.8% after NACT + RT, 90.1% after NACT + CCRT. The 4-year NRFS rates of the three chemoradiotherapy groups were 94.5%, 98.2%, and 97.2%, respectively. Four-year LRRFS rate for ascending type was 84.9% after CCRT, 87.8% after NACT + RT, 87.9% after NACT + CCRT. There was no statistical significance among the difference of the three chemoradiotherapy groups in terms of LRFS, NRFS, and LRRFS (Fig 1C, 1D and 1E; all P > 0.05). The 4-year PFS rates of the three chemoradiotherapy groups were 67.1%, 78.1%, and 77.3%, respectively. Compared with CCRT alone, the PFS of other groups were significantly higher, and this difference had borderline significance among the three groups (Fig 1F; P = 0.049).

**Fig 2:**
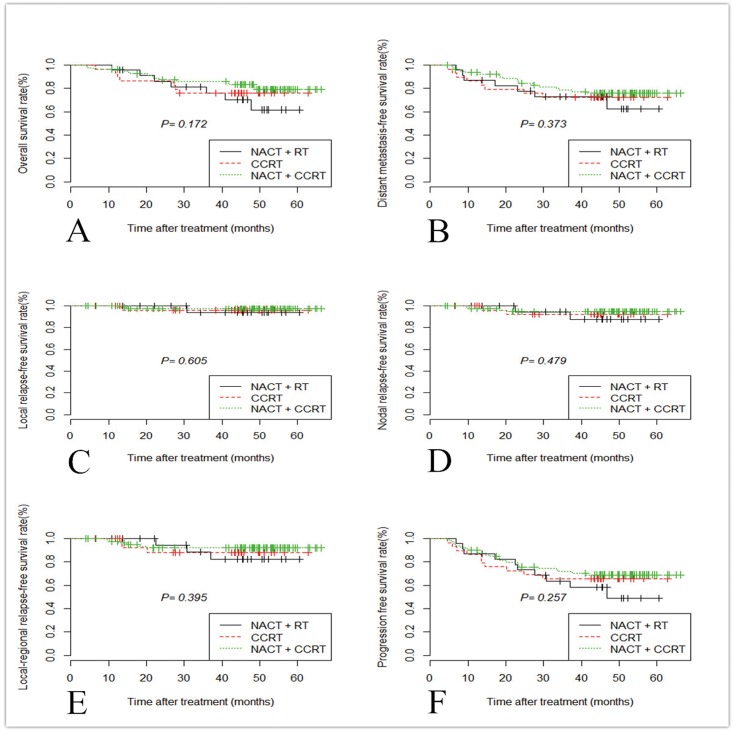
Kaplan-Meier curves of overall survival (A), distant metastasis-free survival (B), local relapse-free survival (C), nodal relapse-free survival (D); loco-regional relapse-free survival (E) and progression-free survival (F) in patients with descending type (T1-2&N3) treated with NACT + RT, CCRT, and NACT + CCRT. *Abbreviations*: NACT: neoadjuvant chemotherapy; RT: radiotherapy; CCRT: concurrent chemoradiotherapy.

For descending type, the 4-year OS rate was 76.7% after CCRT, 69.6% after NACT + RT and 81.3% after NACT + CCRT. Compared with NACT + RT, there was a trend toward a higher OS rate with CCRT and NACT + CCRT, but the correlation did not reach statistical significance (Fig 2A; P = 0.172). The 4-year DMFS rates of the three chemoradiotherapy groups were 73.3%, 66.7%, and 77.5%, respectively. Four-year LRFS rate for the descending type was 96.7% after CCRT, 95.7% after NACT + RT and 97.5% after NACT + CCRT, The 4-year NRFS rates of the three chemoradiotherapy groups were 93.3%, 91.3%, and 95.0%, respectively. Four-year LRRFS rate for descending type was 90.0% after CCRT, 87.0% after NACT + RT and 92.5% after NACT + CCRT, The 4-year PFS rates of the three chemoradiotherapy groups were 66.7%, 56.5%, and 70.0%, respectively. Compared with NACT + RT group, CCRT group and NACT + CCRT group had a better survival rate, while there was no statistical significance among the difference of the three chemoradiotherapy groups (Fig 1B–1F; all P > 0.05).

## Discussion

According to the National Comprehensive Cancer Network, CCRT based on RT is the standard modality for LA-NPC [17]. However, a unified treatment approach may not be appropriate for all patients with LA-NPC. This is the first study to compare the efficacy of neoadjuvant and concurrent chemotherapy in patients with ascending and descending types of NPC treated with IMRT.

### The ascending type

The ascending type of NPC is predominantly advanced local disease with early-stage cervical lymph node involvement. As defined by the 7^th^ UICC/AJCC, patients with T4&N0-1 have intracranial extension and/or cranial nerve involvement into the infratemporal fossa or orbit, and small (< 6 cm) or negative unilateral neck lymph nodes. In the present study, the 4-year OS, DMFS, LRRFS, and PFS rates of the 451 patients with T4&N0-1 NPC were 75.2%, 85.7%, 86.9%, and 73.7%, respectively. Chen *et al*. [18] assessed 154 patients with T4 NPC treated with RT of whom 97% received platinum-based concurrent chemotherapy; 4-year OS, DMFS, LRRFS, and PFS were 78.1%, 72.2%, 81.2%, and 61.9%, respectively. These outcomes are consistent with the OS and LRRFS rates observed in this cohort. However, the proximity of the tumour to critical neural structures in T4 NPC represents a clinical challenge.

Previous studies have reported inconsistent optimal chemotherapy modalities. Xiao *et al*. [2] examined 148 patients with T4 NPC and suggested concurrent chemotherapy was feasible and effective in terms of LRFS (4-year PFS and OS, 46.9% and 75.0%, respectively). In contrast, pooled analysis by Chua *et al*. [19] indicated NACT + RT significantly improved LRRFS and OS in LA-NPC. In this study, NACT + CCRT failed to provide any significant benefit over NACT + RT; concurrent chemotherapy appears to provide no benefit in the ascending type. In addition, NACT + RT significantly improved DMFS and PFS compared to CCRT (4-year DMFS: 90.2% vs. 78.1%, P = 0.004; PFS: 78.1% vs. 67.1%, P = 0.040). One possible explanation is that NACT may effectively control subclinical distant metastatic foci. Furthermore, the significant NACT-induced shrinkage of the primary tumour, which increases the safety margin between the tumour volume and radiation volume, could reduce locoregional recurrence in LA-NPC [20]. In conclusion, concurrent chemotherapy adds no value in terms of local or distant control in patients with the ascending type treated with NACT + RT; NACT + RT may the most reasonable treatment strategy for the ascending type.

### The descending type

The descending type represents advanced lymph node disease with early-stage local invasion. As defined by the 7^th^ UICC/AJCC, patients with T1-2&N3 have metastatic lymph node(s) > 6 cm and/or supraclavicular fossa extension, but the gross tumour is confined to the nasopharynx and/or extends to the parapharyngeal space. The main cause of failure in the descending type is distant metastasis [21–24], and the combination of chemotherapy with radiotherapy is a critical strategy [25–26]. The meta-analysis by Zhang *et al*. [27] demonstrated concurrent chemotherapy was the most effective treatment modality for improving survival. Another meta-analysis by Baujat *et al*. [8] also indicated that the OS benefit could largely be attributed to concurrent chemotherapy in patients with an advanced N classification. Moreover, Yin *et al*. [3] compared different nedaplatin-based chemotherapy regimens in advanced N2-3 category NPC, and demonstrated the benefits of concurrent NFP (nedaplatin, fluorouracil, paclitaxel) chemotherapy in terms of 4-year OS (88.5%) and DMFS (89%).

Nevertheless, CCRT may still not be adequate for certain patients with NPC, especially those with bulky and/or extensive nodal disease at high risk of distant metastasis [28]. Hui *et al*. [9] performed a randomized phase II trial of NACT + CCRT or CCRT alone in stage III-IVb NPC. NACT had a positive impact on survival; 3-year OS was 94.1% for NACT + CCRT versus 67.7% for CCRT (P = 0.012). A recent meta-analysis by Chen *et al*. [10] demonstrated optimizing regimens and identifying patients at high risk of distant metastasis may enhance the efficacy of NACT + CCRT. The current study demonstrates NACT + CCRT could significantly improve OS, DMFS and PFS in the descending type compared to NACT + RT (4-year OS: 81.3% vs. 69.9%, P = 0.021; DMFS: 77.5% vs. 66.7%, P = 0.038; RFS: 70.0% vs. 56.5%, P = 0.021). One explanation for this observation is that CCRT provides OS and DMFS benefits whereas NACT assists with eradication of distant micrometastases and increases the margin of safety between the tumour volume and radiation volume. Therefore, NACT + CCRT may be the most appropriate for the descending type of NPC.

### DMFS for the ascending and descending types

The natural history and failure patterns are quite different between NPC patients with ascending type and descending type. Patients in the former group usually experience local failure while patients in the latter one experience distant failure more often. With the use of IMRT, coupled with the wide adoption of concomitant chemotherapy, the local relapse rate in NPC has significantly decreased, and distant metastases has become to be the predominant model of treatment failures [29]. In the present study, patients with the ascending type had significantly better DMFS than patients with the descending type (4-year DMFS rate: 85.7% vs. 74.1%, P = 0.008). Considering that the advanced N-stage patients had a high distant metastasis rate [21,24,26], this seems to be reasonable because descending type had advanced N classification but early N classification in ascending type.

### OS for the ascending and descending types

Overall, PFS was higher for the ascending type than descending type; this trend was borderline significant (73.7% and 66.2%, respectively; P = 0.063). However, no significant difference in 4-year OS was observed between the ascending and descending types (75.2% and 73.4%, respectively; P = 0.114). One explanation is that the effect of salvage treatment after initial treatment failure cannot be ignored. For example, salvage surgical resection and re-irradiation with external beam radiotherapy have been reported successful in locally recurrent NPC [30–32]. In addition, Chen *et al*. [33] reported a high disease control rate of 93.6% in metastatic and/or recurrent NPC (median follow-up, 24.8 months; median OS, 22.7 months). This may partially explain the borderline significant increase in PFS but absence of a significant difference in OS in the ascending type compared to the descending types.

## Conclusion

In summary, concurrent chemotherapy provides no benefit over IMRT in terms of local recurrence or distant metastasis in ascending type. Compared with NACT + CCRT or CCRT, NACT + RT may be a reasonable approach for ascending type. On the other hand, concurrent chemotherapy was effective in descending type and NACT improved local and distant control, but NACT + CCRT may be a more appropriate strategy for descending type. However, the principal limitation of this study is its retrospective. Therefore, a randomized clinical trial is warranted.
